# Factors influencing JUUL e-cigarette nicotine vapour-induced reward, withdrawal, pharmacokinetics and brain connectivity in rats: sex matters

**DOI:** 10.1038/s41386-023-01773-3

**Published:** 2023-12-07

**Authors:** Jude A. Frie, Patrick McCunn, Amr Eed, Ahmad Hassan, Karling R. Luciani, Chuyun Chen, Rachel F. Tyndale, Jibran Y. Khokhar

**Affiliations:** 1 Department of Biomedical Sciences, Ontario Veterinary College, University of Guelph, Guelph, ON Canada; 2 Department of Anatomy and Cell Biology, Schulich School of Medicine and Dentistry, Western University, London, ON Canada; 3 Department of Medical Biophysics and Robarts Research Institute, Schulich School of Medicine and Dentistry, Western University, London, ON Canada; 4 Department of Psychiatry, Faculty of Medicine, University of British Columbia, Vancouver, BC Canada; 5 Departments of Psychiatry, and Pharmacology & Toxicology, University of Toronto, Campbell Family Mental Health Research Institute, Centre for Addiction and Mental Health, Toronto, ON Canada

**Keywords:** Reward, Motivation

## Abstract

Though vaping likely represents a safer alternative to smoking, it is not without risks, many of which are not well understood, especially for vulnerable populations. Here we evaluate the sex- and age-dependent effects of JUUL nicotine vapour in rats. Following passive nicotine vapour exposures (from 59 mg/ml JUUL nicotine pods), rats were evaluated for reward-like behaviour, locomotion, and precipitated withdrawal. Pharmacokinetics of nicotine and its metabolites in brain and plasma and the long-term impact of nicotine vapour exposure on functional magnetic resonance imaging-based brain connectivity were assessed. Adult female rats acquired conditioned place preference (CPP) at a high dose (600 s of exposure) of nicotine vapour while female adolescents, as well as male adults and adolescents did not. Adult and adolescent male rats displayed nicotine vapour-induced precipitated withdrawal and hyperlocomotion, while both adult and adolescent female rats did not. Adult females showed higher venous and arterial plasma and brain nicotine and nicotine metabolite concentrations compared to adult males and adolescent females. Adolescent females showed higher brain nicotine concentration compared to adolescent males. Both network-based statistics and between-component group connectivity analyses uncovered reduced connectivity in nicotine-exposed rats, with a significant group by sex interaction observed in both analyses. The short- and long-term effects of nicotine vapour are affected by sex and age, with distinct behavioural, pharmacokinetic, and altered network connectivity outcomes dependent on these variables.

## Introduction

Nicotine use has seen drastic changes in recent years, with steady declines in smoking and increases in electronic cigarette nicotine vapour use (vaping) [1]. Although vaping likely represents a safer alternative to smoking [2], many unknowns regarding its effects remain, especially for never-smokers [3]. There is also specific concern among populations vulnerable to nicotine’s negative effects, such as adolescents and women [4, 5].

Adolescent vaping remains high, with over 2 million youth reporting past 30-day e-cigarette use in the US [1]. This is concerning, as adolescent nicotine use has been associated with increased risk of negative behavioural outcomes, including cognitive impairment, psychiatric and mood disorders, and future substance use [6]. Adolescents who vape are also more likely to transition to combustible cigarettes [7].

Women also present a population vulnerable to nicotine, with lower smoking cessation rates and higher risk of tobacco-related morbidity and mortality [8, 9]. Adult women use double the nicotine concentration when vaping, show signs of greater e-cigarette dependence, and are more likely to transition to cigarettes from vaping than men [10, 11].

One brand that has contributed to the rapid uptake of e-cigarettes is JUUL, which introduced higher nicotine, salt-based pods [12, 13]. Their use was disproportionately higher in youth and young adults [14], including under-age users [15], and known to produce higher nicotine concentrations in users, even compared to combustible cigarettes [16, 17]. Importantly, youth found JUUL rewarding [18], and the high-dose nicotine pods (59 mg/ml) produced high liking and wanting as well as urge to use [19].

Preclinical studies have found robust age-dependent differences in nicotine pharmacokinetics, reinforcement, reward, and withdrawal with systemic nicotine exposure. Adolescent rats show greater nicotine volume of distribution and 2-fold increased plasma clearance following intravenous nicotine injection [20]. Adolescent rats have been shown to self-administer more nicotine during intravenous self-administration (IVSA) and administer more nicotine than adults [21, 22], and at lower doses when exposed to nicotine in adolescence [23]. Conversely, some studies have shown increased sensitivity to IVSA and relapse in adults compared to adolescents [24, 25]. Therefore, before any conclusive statements regarding adolescent sensitivity to nicotine may be made, more studies investigating this sensitivity are required. Adolescent rats display greater conditioned place preference (CPP) than adults and at lower doses regardless of route of administration [26–28]. During precipitated withdrawal from nicotine administered via osmotic minipump, male adolescents show reduced signs of withdrawal compared to adults and no withdrawal-induced anxiety-like behaviour in elevated plus maze (EPM) [29–31].

Significant preclinical sex differences in nicotine pharmacokinetics and behaviour have also been seen. Female rats show higher plasma levels following repeated exposures [32], and reduced rates of nicotine metabolism [33]. Female rats acquire nicotine IVSA faster, at lower doses, and administer more nicotine than males [34, 35]. Female rats require higher doses to acquire CPP compared to males [36], or do not acquire CPP at examined doses [37, 38]. These findings suggest a right-shifted dose-response curve to nicotine CPP in female rodents [39]. Female rats also do not display signs of precipitated nicotine withdrawal [40], but do show more anxiety-like behaviour in an EPM and higher corticosterone levels compared to males during withdrawal [41].

Though injections and osmotic mini-pumps have shown utility for understanding the effects of nicotine on the brain, the unique characteristics associated with nicotine e-liquid’s additional constituents and pulmonary exposure route warrants investigation. Vapour models allow for the study of e-cigarette use parameters otherwise not possible, such as flavours, wattage, and salt/base ratio. Previous vapour findings have also shown that rodents will self-administer nicotine vapour [42, 43], that nicotine vapour exposure leads to robust signs of physical dependence in adult male rats [44], and that nicotine vapour self-administration is potentiated by flavours [45, 46], and nicotine salt [47]. Female rats also show greater increases in approach to the nicotine delivery port, and in adolescence, female nose pokes for nicotine vapour correlate with anxiety-like behaviour in the EPM [48]. Thus, preclinical nicotine vapour-based models provide a powerful tool for uncovering sex- and age-dependent vulnerabilities to nicotine.

In the present study, we aimed to extend our previous findings showing increased nicotine vapour CPP in adolescent male rats to determine if female adolescents are similarly vulnerable [28]. We also investigate sex- and age-dependent effects of nicotine vapour on withdrawal-like behaviour and nicotine pharmacokinetics. Finally, using functional MRI, we determine the impact of repeated nicotine vapour exposure on network connectivity.

## Materials and methods

### Animals

Sprague Dawley rats (Charles River Lab, St. Constant, Canada) were kept on a 12:12 light-dark cycle (lights off at 0800 h) in a colony maintained at 21 °C housed in groups of 3-4. Food (Envigo, Madison, Wisconsin, USA, Rodent Diet, 14% protein) and water were available *ad libitum*. Adolescent rats arrived with dams on post-natal day (PND) 21 and were weaned on PND 23. Rats were handled for two minutes daily for 3 days before the start of experiments. For place conditioning, 7 adult males, 7 adolescent males, 35 adult females and 35 adolescent females were used. Three sets of place conditioning experiments were conducted in separate cohorts: 1) Female adults (VEH, 120, 240, and 480 s; *n* = 7/treatment), 2) Female adolescents (VEH, 120, 240, and 480 s; *n* = 7/treatment), and 3) Male or female adults and adolescents (600 s; *n* = 7/sex/age). For withdrawal and locomotion experiments, 16 adult males, 14 adolescent males, 16 adult females, and 14 adolescent females were used, with each group run in separate cohorts. For the pharmacokinetic studies, 14 rats per sex and age were used, with each group of exposures run in separate cohorts, but all samples were analysed together. For MRI 20 rats per sex and age were used, with males and females run in separate cohorts. All experiments were conducted in the light cycle. All procedures were performed in accordance with the University of Guelph Animal Care Committee, the University of Western Ontario Animal Use Subcommittee, and were consistent with guidelines established by the Canadian Council on Animal Care.

### Reagents

Vapour exposures used either mint nicotine JUUL pods (30:70 PG:VG; 59 mg/ml nicotine; JUUL Labs, Washington D.C., United States) or mint vehicle JUUL pods (30:70 PG:VG, 10% mint flavour; E-Cigz vape shop, Guelph, ON). Mecamylamine hydrochloride (1.5 mg/kg in saline; Sigma-Aldrich) was used for withdrawal experiments.

### Place conditioning

Vapour exposures were conducted in OpenVape [28]. For place conditioning, each group spent 600 s in the vapour chamber while receiving 120, 240, 480 or 600 s of pump activation resulting in varying cloud thicknesses. Each epoch of pump activation produced a 2-s puff duration, followed by a 4-s time-out resulting in ten 2-s puffs/min. Control groups received vehicle vapour for 480 s. Place conditioning was performed as published previously [28].

### Withdrawal

Rats were exposed for 600 s, three times a day, 6 h apart, for 14 days via OpenVape starting on PND 30 for adolescents and PND 74 for adults. Sixteen hours following their final exposure, they received a 1.5 mg/kg intraperitoneal injection of mecamylamine and were placed in a 30 × 30 cm Pyrex cylinder. 20 min following injections, rats were monitored for signs of withdrawal for 10 min. To ensure that mecamylamine did not produce withdrawal symptoms on its own in the control group, a lower dose was used to precipitate withdrawal at the start of the spontaneous withdrawal window [49]. Signs measured included teeth chattering, headshakes, yawns, ptosis, foot licks, writhes, cheek tremors, eye blink, body shake, chews, gasps, tremors, grooming, and escape attempts [29, 50]. Subjects were habituated to the cylinders for 2 days (10 min each day). Withdrawal was recorded using a GoPro Hero 8. Withdrawal signs were counted by two blinded scorers whose results were averaged and used as the final withdrawal score.

### Locomotion

Locomotion was measured at 5 time points using Ethovision. Rats were habituated to the open field (50 × 50 cm) for 15 min the day prior to taking baseline. Following the second vapour exposure of the day on exposure days 1, 7, 11, and 14, locomotion was measured for 1-h.

### Pharmacokinetics

After one 600-s vapour exposure (as done in CPP and withdrawal experiments), saphenous blood draws were conducted for each rat. Trunk blood and brains were then collected at either 30- or 60-min following exposure initiation. Plasma was collected after centrifugation at 3500 *g* for 10 min. Brain samples (whole brain) were homogenised in three volumes ice-cold saline (0.9% NaCl) and centrifuged at 3000 *g* for 10 min and supernatant collected. Liquid chromatography with tandem mass spectrometry analysis quantified plasma and brain supernatant levels of nicotine, cotinine, nornicotine, 3-hydroxycotinine, nicotine-N′-oxide, and norcotinine following previously published sample preparation and quantification methods [20].

### Magnetic resonance imaging

Rats were exposed for 600 s, three times a day, 6 h apart, for 14 days (PND 30–43 for adolescents, PND 74–87 for adults) via OpenVape [28]. Scans were performed after a minimum washout period of 14 days. For scans, as before [51], rats were placed in an induction chamber with 4–5% isoflurane and an oxygen flow rate of 1–1.5 L/min. Following induction, isoflurane was maintained between 2.0% and 2.5% with an oxygen flow rate of 1–1.5 L/min through a nose cone and an intraperitoneal injection of 0.018 mg/kg dexmedetomidine was administered. Once the rat was positioned in the MRI scanner, a constant rate infusion of 0.018 mg/kg/h dexmedetomidine was initiated for the duration of the experiment [52]. Following initiation of the dexmedetomidine infusion, isoflurane was reduced over a 15-min period from 2.0–2.5% to 0.8–1.0% with an oxygen flow rate of 1–1.5 L/min to maintain optimal physiology (respiration rate: 74 ± 5.2 breaths per minute; heart rate: 364 ± 23 beats per minute). The rectal temperature was kept at 37.0 ± 0.5 °C with an air heater. As in previous publications, the EPI sequences were initiated when optimal physiological conditions were reached [51, 52].

### Acquisition

Images were acquired using a 9.4 T Bruker small animal MRI scanner at the Centre for Functional and Metabolic Mapping located within Robarts Research Institute at Western University.

### Anatomical

T2 Anatomical images were acquired for each subject at the beginning of each session with T2-weighted TurboRARE pulse sequence [53] (8 averages, 35 slices, slice thickness = 400 μm, FOV 38.4 × 38.4 mm, matrix size 192 × 192, in-plane resolutio*n* = 200 × 200 μm, TE = 44.0 ms, TR = 7.0 s, Echo Spacing = 11.00 ms, Rare Factor 8, total acquisition time = 14 min).

### Functional

Resting-state fMRI images were acquired based on optimised scan parameters [54], using a gradient-echo EPI sequence (2 runs, 400 volumes per run, TE = 15.0 ms, TR = 1.5 s, FOV 38.4 × 38.4 mm, matrix size 96 × 96, 35 slices, isotropic resolutio*n* = 400 μm, bandwidth 280 kHz).

### Diffusion

Diffusion images were acquired using a multi-shot, spin echo, echo-planar-imaging (EPI) acquisition pulse sequence (4 shots, 32 slices, slice thickness = 500 μm, FOV 40 × 40 mm, matrix size, 160 × 160, in-plane resolutio*n* = 250 × 250 μm, TE = 26.71 ms, TR = 2.5 s). The diffusion scheme used was previously described in detail and was shown to produce reproducible and reliable results at 9.4 Tesla [55]. (Four Averages, Shell one: 30 directions, *b* value = 1000 s/mm^2^, Shell two: 60 directions, *b* value = 2000 s/mm^2^, ten *b* = 0 s/mm^2^ shells were interspersed evenly throughout the acquisition).

### Image processing

Processing was conducted using RABIES software and the SIGMA rat brain template [56, 57]. Please refer to the supplementary methods for a detailed description of the image processing pipeline.

### Statistical analysis

Statistics were conducted using IBM SPSS Statistics 26. For CPP, a conditioning index was calculated as the difference in time spent in the initially preferred chamber pre- and post-conditioning. Outliers were identified as those whose conditioning index lied 2 SD from the mean. One female adolescent was identified as an outlier. Determination of CPP was by Bonferroni corrected one-sample t-tests against a hypothetical conditioning index of zero for each group [58]. Sex and age differences at the 10-min dose were compared via two-way ANOVA with age and sex as between subject factors.

Total withdrawal scores were analysed via three-way ANOVA with age, sex, and treatment as between subject factors followed by planned post hoc comparisons using Fisher’s LSD. Locomotion and weight were analysed by four-way ANOVA with age, sex, and treatment as between subject factors and day as a repeated measure. Significant interactions were followed by separate two-way ANOVA and post hoc Bonferroni comparisons.

Venous plasma nicotine levels were compared via a two-way ANOVA with age and sex as between subject factors followed by Tukey post hoc multiple comparisons. Arterial plasma nicotine levels were analysed by three-way ANOVA with age, sex, and time point as between subject factors followed by one-way ANOVA comparing all groups at each time point with Tukey post hoc multiple comparisons. Metabolite’s measurements that were below the 1 ng/mL limit of quantification were removed.

For MRI studies, the Network-based statistic (NBS) was used to identify significantly different subnetworks between groups [59]; network-based models have recently been postulated to facilitate cross-species translation [60], which was a primary objective behind our imaging studies. Unthresholded correlated matrices were used in NBS to preserve the full range of connectivity information and avoid introducing confounding effects and bias that may result when multiple measures of connectivity are being assessed [59]. Two tailed t-tests were run for main effects of group (*p* = 0.05) with age and sex as covariates. Significant networks were extracted for analysis of interaction effects. Group by age and group by sex interaction were tested using an f-test with Bonferroni correction (*p* = 0.025).

A complementary between-component group analysis was also conducted. The preprocessed EPI time-series output from RABIES was smoothed using a FWHM Gaussian kernel of 0.6 mm. High-pass filtering using a cut-off of 100 s was then performed to remove the low-frequency noise and to preserve the signal associated with resting-state networks (>0.01 Hz). The 4D images of all subjects were then concatenated and group independent-component analysis (ICA) was performed using FSL’s MELODIC. The concatenated data was decomposed into 30 dimensions. Seven networks were classified as noise and were not considered for any subsequent analysis. To assess between-component connectivity strengths, FSLNets was used; the full correlation between each pair of nodes was calculated and converted to *z*-scores. Non-parametric permutation testing (10,000 permutations) was used to test for group differences between nicotine intake and vehicle using sex as a covariate which was tested for positive and negative sex interactions.

## Results

### Place conditioning

Place conditioning results suggested an increase in reward-like responses in female adult rats to higher doses of nicotine vapour, while this dose did not produce CPP in adolescent females, as well as adult and adolescent males.

**Fig. 1:**
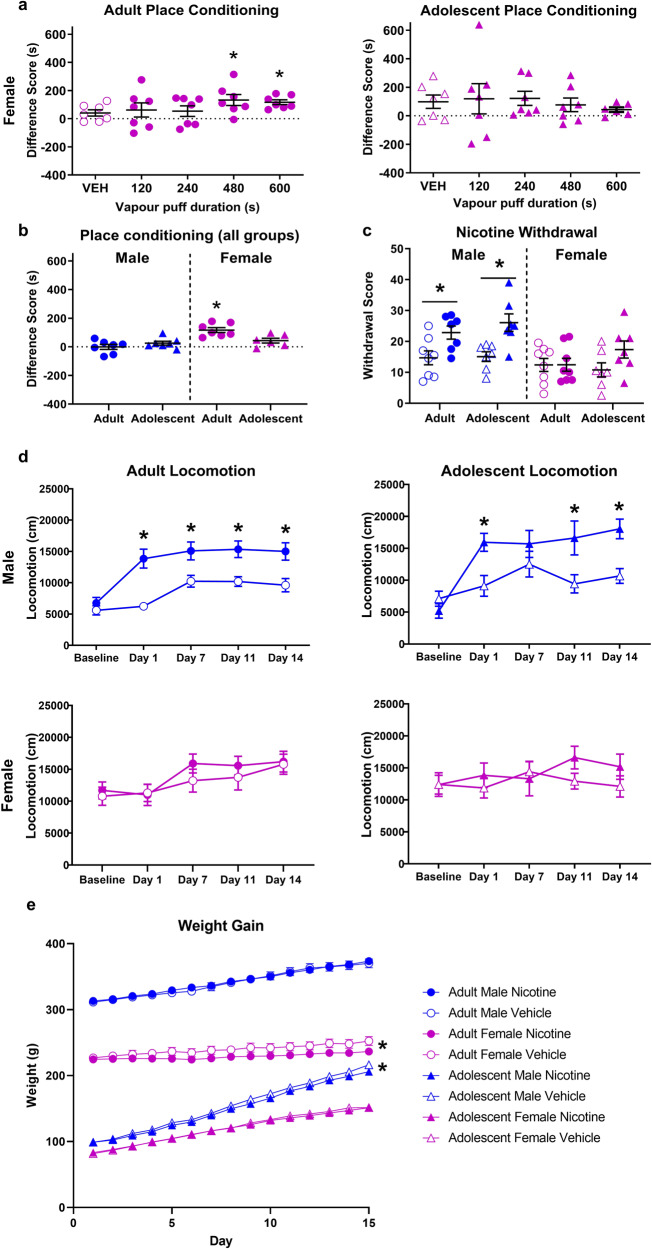
Sex and age impact the effects of nicotine vapour on reward, withdrawal, locomotion and body weight. **a** Female nicotine vapour place conditioning results. Difference score is defined as the difference in time spent in the initially non-preferred chamber pre- to post-conditioning. Adult females acquire CPP at 480 and 600 s doses. Adolescent females do not acquire CPP at any dose tested. **b** Nicotine vapour place conditioning results for male and female, adult, and adolescent rats at 600 s dose. *p < 0.05 significant shift in preference from zero. **c** Nicotine (filled circles) and vehicle (empty circles) vapour withdrawal scores. Scores are the sum of all observed somatic withdrawal signs averaged across two blinded scorers. Male adults and adolescents show similar levels of nicotine vapour withdrawal signs compared to control. Females do not show significant levels of nicotine vapour withdrawal though adolescents appear to trend toward significance. *p < 0.05 nicotine vapour versus vehicle vapour. **d** Nicotine (filled circles) and vehicle (empty circles) vapour effect on locomotion. Locomotion was measured following the second exposure of the day. Adult and adolescent males show significantly higher locomotion following nicotine vapour exposure. Adult and adolescent females do not show any increase in locomotion in response to nicotine vapour exposure. *p < 0.05 nicotine vapour versus vehicle vapour. **e** Weight change over nicotine vapour treatment period. Adolescent males and adult females have reduced weight gain in response to repeated nicotine vapour exposure. *p < 0.05 significant treatment by day interaction. Data are presented as mean ± SEM.

Bonferroni corrected t-tests confirmed significant CPP for adult females at 480 (t(7) = 3.424, *p* = 0.0352) and 600 (t(7) = 6.831, *p* = 0.0012) s (Fig. 1a). There was no significant preference at any dose in adolescent females.

Two-way ANOVA confirmed a significant effect of sex (F(1,23) = 17.20, *p* = 0.0004) but not age (F(1,23) = 2.117, *p* = 0.1592), and a significant interaction (F(1,23) = 9.441, *p* = 0.0054). Post hoc further confirmed significantly greater CPP in adult females compared to adolescent females (*p* = 0.0273), adult males (*p* = 0.0002), and adolescent males (*p* = 0.003; Fig. 1b).

### Withdrawal

Significant nicotine withdrawal was seen in adult and adolescent males, but not females of either age.

Three-way ANOVA confirmed significant main effects of treatment (F(1,51) = 15.99, *p* = 0.0002) and sex (F(1,51) = 15.91, *p* = 0.0002). Post hoc further confirmed more withdrawal signs in nicotine-exposed adult males and adolescent males versus control (t(51) = 2.553, *P* = 0.0137 and t(51) = 3.329, *p* = 0.0016 respectively) and female nicotine rats (t(51) = 3.257, *p* = 0.0020 and t(51) = 2.637, *p* = 0.0111 respectively; Fig. 1c).

### Locomotion

Both adult and adolescent males showed increased locomotor activity in response to nicotine vapour treatment. Females did not show increased activity but did have higher locomotion at baseline.

Four-way ANOVA confirmed a between-subjects effects of treatment (F(1,49) = 10.601, *p* = 0.002) and sex (F(1,49) = 4.742, *p* = 0.034) and within-subjects effect of day (F(4,196) = 34.979, *p* < 0.0001) with day by treatment (F(4,196) = 5.810, *p* = 0.000194), day by sex (F(4,196) = 7.189, *p* < 0.0001), and day by treatment by sex (F(4,196) = 2.903, *p* = 0.023) interactions (Fig. 1d).

Two-way ANOVA in adult males confirmed an effect of day (F(4,56) = 45.58, *p* < 0.0001), treatment (F(1,14) = 12.37, *p* = 0.0034), and day by treatment (F(4,56) = 7.802, *p* < 0.0001) interaction. Post hoc further confirmed higher locomotion in nicotine treated rats on day 1 (t(70) = 4.873, *p* < 0.0001), 7 (t(70) = 3.102, *p* = 0.0138), 11 (t(70) = 3.297, *p* = 0.0077), and 14 (t(70) = 3.443, *p* = 0.0049). Two-way ANOVA on adolescent males confirmed significant day (F(4,43) = 20.23, *p* < 0.0001), treatment (F(1,12) = 5.918, *p* = 0.0316), and day by treatment (F(4,43) = 6.879, *p* = 0.0002) interaction. Post hoc confirmed higher locomotion in nicotine-treated rats on day 1 (t(55) = 3.054, *p* = 0.0174), 11 (t(55) = 2.953, *p* = 0.0231), and 14 (t(55) = 3.048, *p* = 0.0177). Two-way ANOVA on adult females confirmed an effect of day (F(4,56) = 14.56, *p* < 0.0001), and adolescent females showed no significant differences at any point.

### Weight

Adolescent males and adult females gained less weight over the exposure period compared to controls.

Four-way ANOVA confirmed significant between-subjects effects of sex (F(1,52) = 488.839, *p* < 0.0001) and age (F(1,52) = 2164.035, *p* < 0.0001), with a sex by age interaction (F(1,52) = 136.062, *p* < 0.0001). There were also within-subjects effects of day (F(13,676) = 2020.853, *p* < 0.0001), day by treatment (F(13,676) = 3.923, *p* = 0.010), day by sex (F(13,676) = 248.339, *p* < 0.0001), day by age (F(13,676) = 329.698, *p* < 0.0001), and day by sex by age (F(13,676) = 3.374, *p* = 0.020). Two-way ANOVA on each group confirmed a treatment by day interaction in adult females (F(14,196) = 5.447, *p* < 0.0001) and adolescent males (F(14,168) = 2.198, *p* = 0.0097; Fig. 1e).

### Pharmacokinetics

Female adults had greater venous nicotine and cotinine levels than adolescent females at the 10-minute timepoint. Moreover, female adults also had greater arterial plasma nicotine and metabolite concentrations compared to adult males.

### Venous blood plasma

**Fig. 2:**
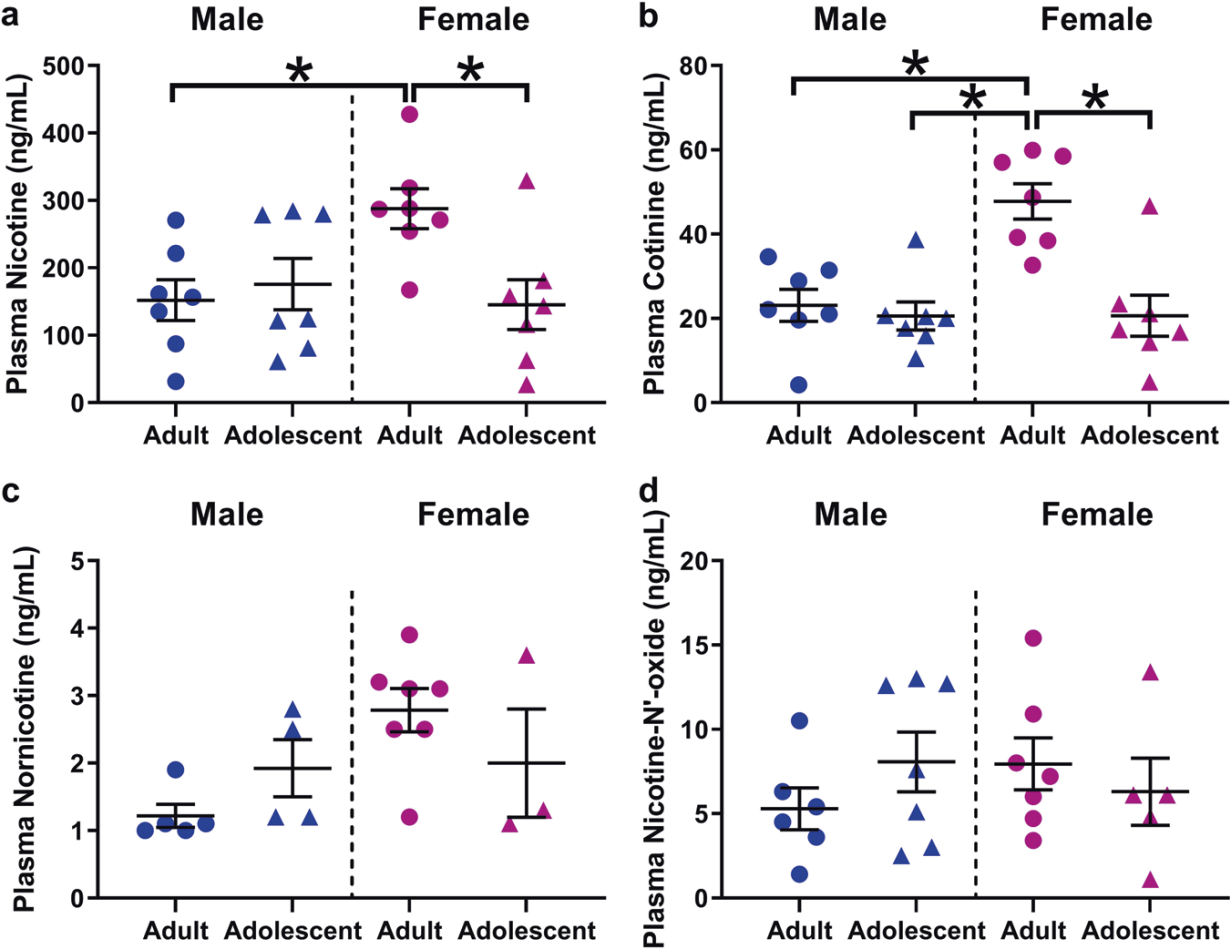
Higher venous plasma nicotine levels in female adults compared to adolescents and male rats. **a** comparison of age and sex on venous nicotine plasma level. Adult females display greater nicotine levels compared to adult males or adolescent females. **b** comparison of age and sex on venous cotinine plasma level. Adult females display greater cotinine levels than all other groups. **c** Comparison of age and sex on venous nornicotine plasma level. **d** comparison of age and sex on venous nicotine-n’-oxide plasma level. *p < 0.05 adult versus adolescent or male versus female. *N* = 7 per group and timepoint. Data are presented as mean ± SEM.

Venous plasma nicotine levels are presented in Fig. 2a. Two-way ANOVA confirmed a significant interaction between age and sex (F(1,24) = 6.021, *p* = 0.0218). Post hoc analyses showed greater nicotine in adult females compared to adult males (*p* = 0.0424), or adolescent females (*p* = 0.0313).

Venous plasma cotinine levels are presented in Fig. 2b. Two-way ANOVA confirmed a significant effect of sex (F(1,24) = 9.125, *p* = 0.0059), age (F(1,24) = 13.16, *p* = 0.0013), and sex by age interaction (F(1,24) = 9.019, *p* = 0.0062). Post-hoc showed greater plasma cotinine in adult females compared to adult males (*p* = 0.0015), adolescent females (*p* = 0.0005), and adolescent males (*p* = 0.0005). No significant differences were seen in nornicotine (Fig. 2c) or nicotine-N’-oxide levels (Fig. 2d). All other metabolites were too low for quantification and comparison at this time point.

### Arterial blood plasma

**Fig. 3:**
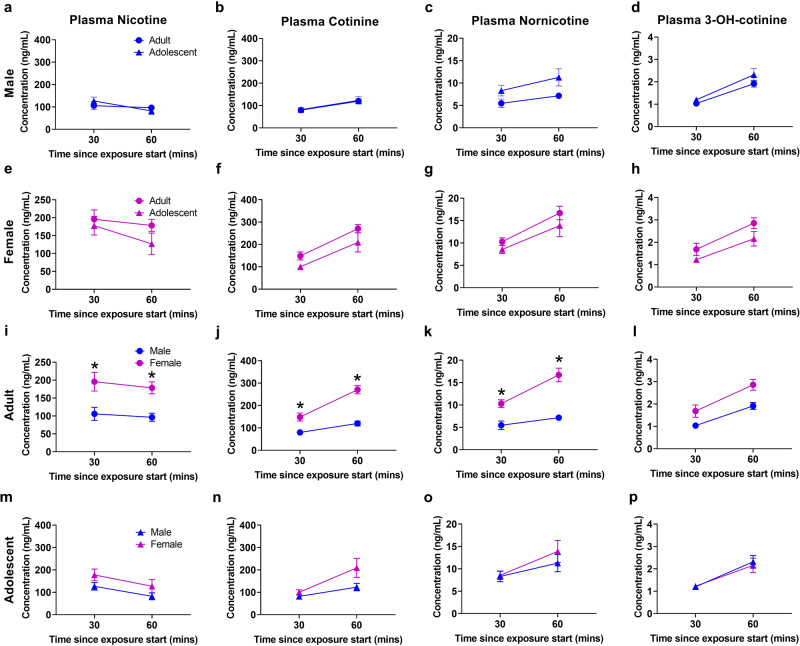
Higher arterial plasma nicotine levels in female adults compared to male adult rats. **a–d** Comparison of adult to adolescent males. **e–h** Comparison of adult to adolescent females. **i–l** Comparison of adult males to adult females. Adult females display greater nicotine, cotinine, and nornicotine compared to adolescent females. **m–p** Comparison of adolescent males to adolescent females. *p < 0.05 adult versus adolescent or male versus female. *N* = 7 per group and timepoint. Data are presented as mean ± SEM.

Arterial plasma nicotine pharmacokinetic data are presented in Fig. 3a, e, i, and m. Three-way ANOVA of nicotine plasma confirmed significant effects of time (F(1,48) = 4.236, *p* = 0.0450) and sex (F(1,48) = 20.33, *p* < 0.0001). Post hoc showed greater plasma nicotine in adult females versus adult males at 30 (*p* = 0.0406), and 60 (*p* = 0.0330) minute time points.

Arterial plasma cotinine pharmacokinetic data are presented in Fig. 3b, f, j, and n. Three-way ANOVA showed effects of time (F(1,48) = 30.50, *p* < 0.0001) and sex (F(1,48) = 32.66, *p* < 0.0001), as well as time by sex (F(1,48) = 6.964, *p* < 0.0112), and sex by age (F(1,48) = 4.164, *p* = 0.0468) interactions. Post hoc showed greater plasma cotinine levels in female adults versus male adults at 30 (*p* = 0.0048) and 60 (*p* = 0.0016) minutes.

Arterial plasma nornicotine pharmacokinetic data are presented in Fig. 3c, g, k, and o. Three-way ANOVA confirmed an effect of time (F(1,48) = 16.55, *p* = 0.0002), sex (F(1,48) = 18.26, *p* < 0.0001), and sex by age (F(1,48) = 8.157, *p* = 0.0063) interaction. Post hoc showed greater plasma nornicotine concentrations in adult females versus adult males at 30 (*p* = 0.0107) and 60 (*p* = 0.0040) min.

Arterial plasma 3-hydroxycotinine pharmacokinetic data can be seen in Fig. 3d, h, l, and p. No significant differences were found between groups.

Arterial plasma nicotine-N′-oxide pharmacokinetic data can be seen in Supplementary Fig. 1a, c, e, and g. Three-way ANOVA confirmed an effect of time (F(1,48) = 63.81, *p* < 0.0001), sex (F(1,48) = 45.11, *p* < 0.0001), and age (F(1,48) = 15.50, *p* = 0.0003), as well as time by sex (F(1,48) = 36.67, *p* < 0.0001), time by age (F(1,48) = 29.01, *p* < 0.0001), sex by age (F(1,48) = 55.38, *p* < 0.0001), and time by sex by age (F(1,48) = 35.72, *p* < 0.0001) interactions. Post hoc showed greater plasma levels in adult females versus adult males (*p* < 0.0001), and adolescent females (*p* < 0.0001).

Arterial plasma norcotinine levels can be seen in Supplementary Fig. 1b, d, f, and h. Three-way ANOVA confirmed an effect of time (F(1,47) = 43.45, *p* < 0.0001) and sex (F(1,47) = 32.59, *p* < 0.0001), as well as time by sex (F(1,47) = 6.457, *p* < 0.0144), and sex by age (F(1,47) = 6.457, *p* < 0.0144) interactions. Post hoc showed greater plasma levels in adult females versus adult males (*p* < 0.0001), and adolescent females (*p* < 0.0001) at 60 min.

### Brain

Overall, adult and adolescent females had higher brain nicotine and metabolite levels than adult and adolescent males, respectively.

**Fig. 4:**
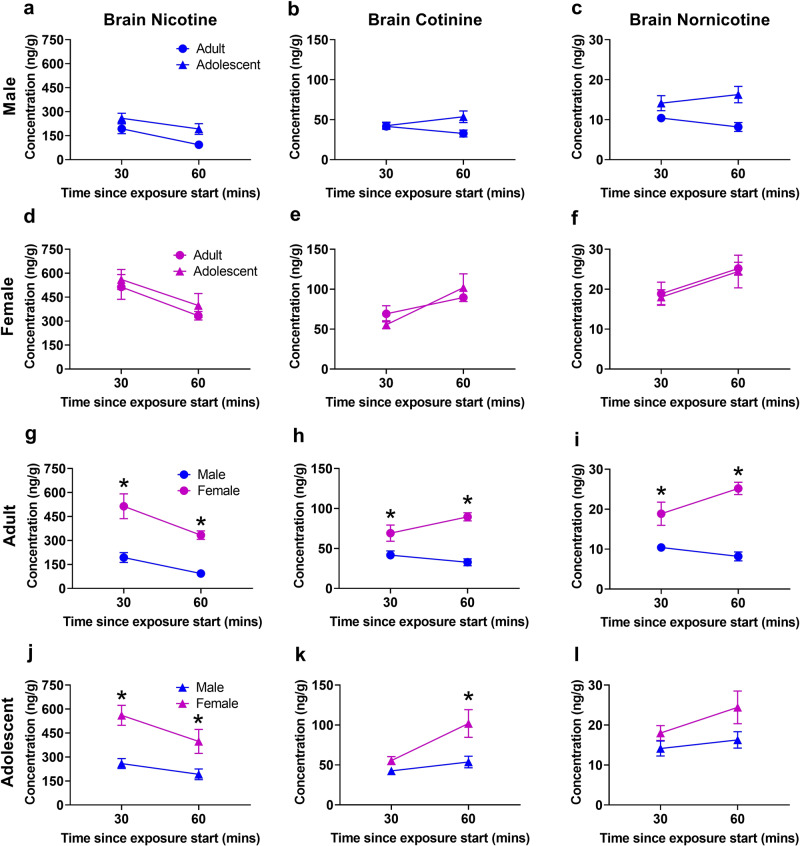
Higher brain nicotine levels in female adult and adolescent rats compared to male rats. **a–c** Comparison of adult males to adolescent males. **d–f** Comparison of adult females to adolescent females. **g–i** Comparison of adult males to adult females. Adult females display greater nicotine, cotinine, and nornicotine concentrations compared to adult males. **j–l** Comparison of adolescent males to adolescent females. Adolescent females display higher nicotine and cotinine concentrations compared to adolescent males. *p < 0.05 adult versus adolescent or male versus female. *N* = 7 per group and timepoint. Data are presented as mean ± SEM.

Nicotine brain pharmacokinetics are presented in Fig. 4a, d, g, and j. Three-way ANOVA confirmed effects of time (F(1,48) = 13.44, *p* = 0.0006) and sex (F(1,48) = 58.65, *p* < 0.0001). Post hoc showed higher nicotine in adult females versus adult males at 30 (*p* = 0.0018), and 60 (*p* = 0.0036) min as well as greater concentrations in adolescent females versus adolescent males at 30 (*p* = 0.0033) and 60 (*p* = 0.0137) min.

Cotinine brain pharmacokinetics are presented in Fig. 4b, e, h, and k. Three-way ANOVA showed main effects of time (F(1,48) = 8.463, *p* = 0.0055) and sex (F(1,48) = 37.51, *p* < 0.0001), and a time by sex interaction (F(1,48) = 7.425, *p* = 0.0089). Post hoc analysis showed greater concentrations in adult females compared to adult males at 30 (*p* = 0.0291) and 60 (*p* = 0.0026) min and greater concentrations in adolescent females versus adolescent males at 60 (*p* = 0.0112) min.

Nornicotine brain pharmacokinetics are presented in Fig. 4c, f, i, and l. Three-way ANOVA confirmed an effect of sex (F(1,48) = 34.42, *p* < 0.0001) as well as time by sex (F(1,48) = 4.056, *P* = 0.0496) and sex by age (F(1,48) = 4.443, *p* = 0.0403) interactions. Post hoc showed greater brain nornicotine concentrations in adult females versus adult males at 30 (*p* = 0.0320) and 60 (*p* = 0.0003) min.

Brain levels of 3-hydroxycotinine, nicotine-N’-oxide, and nornicotine were below the limit of quantification at the selected time points.

### Magnetic resonance imaging

In all groups, NBS confirmed reduced functional connectivity in the nicotine group versus their respective vehicle controls. This effect was more pronounced in female compared to male rats.

**Fig. 5:**
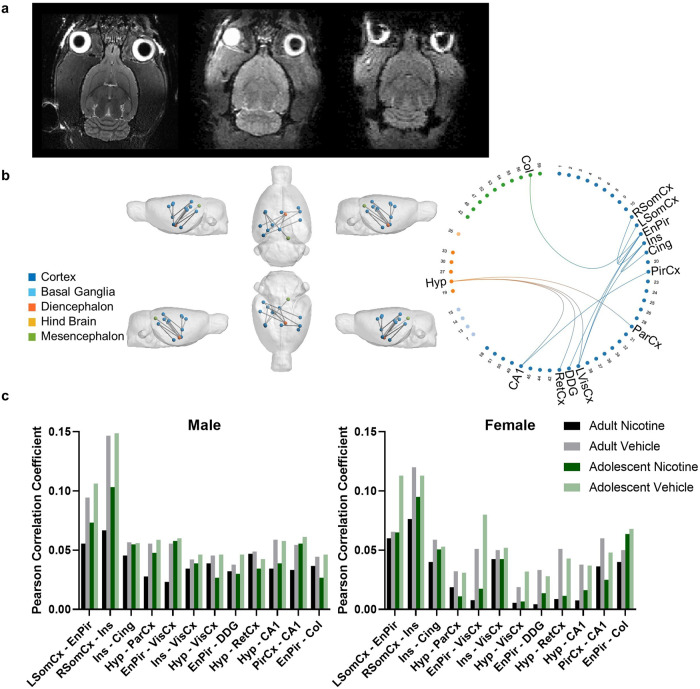
Reduced functional connectivity was observed in nicotine vapour-exposed rats, with greater reductions observed in female rats. **a** Representative single subject T2 anatomical image (left), diffusion b = 0 image (middle), and the first volume of an fMRI dataset (right). **b** NBS statistics confirmed reduced functional connectivity in the Nicotine group (*n* = 34) compared to the Vehicle group (*n* = 36) when controlling for age and sex (*p* = 0.013, 12 edges, 13 nodes). Significant edges have anatomical regions labelled. All other regions are labelled with their numerical SIGMA atlas reference. **c** Average Pearson Correlation Coefficients in edges identified by NBS statistics to have reduced functional connectivity in the Nicotine group when controlling for age and sex (*p* = 0.013, 12 edges, 13 nodes). Post-hoc analysis confirmed a statistically significant group by sex interaction effect (*p* < 0.001, 5 edges, 6 nodes). No statistically significant group-by-age interaction effect was confirmed. Abbreviations: L Left, R Right, Hyp Hypothalamus, ParCx Parietal Cortex, PirCx Piriform Cortex, EnPir Endo/piriform Cortex, VisCx Primary and Secondary Visual Cortex, CA1 Cornu Ammonis 1, SomCx Primary Somatosensory Cortex, Ins Insular Cortex, Cing Cingulate Cortex, DDG Dorsal Dentate Gyrus, RetCx Retrosplenial Cortex, Col Colliculus.

Data was successfully collected from 78 rats. Figure 5a (left) shows a representative single-subject T2 anatomical image and the first volume of an fMRI dataset (right).

Eight data sets were identified that contained motion-related and slice timing artifacts which could not be adequately corrected with preprocessing and were subsequently removed, leaving *n* = 70 datasets available for analysis (Table 1). Commonly studied resting networks were conserved in the functional datasets (Supplementary Fig. 2).

**Table 1:** Subjects.

Group		Sex	n	Age ± SD (Days)	Weight ± SD (g)
Nicotine	Adult	Male	9	111 ± 9	488 ± 42
		Female	8	138 ± 4	426 ± 53
	Adolescent	Male	9	65 ± 3	323 ± 17
		Female	9	59 ± 9	308 ± 27
Vehicle	Adult	Male	9	121 ± 7	493 ± 67
		Female	8	132 ± 6	447 ± 22
	Adolescent	Male	8	59 ± 8	366 ± 23
		Female	10	68 ± 11	297 ± 64

In the Nicotine group, NBS statistics found a single network with reduced functional connectivity when controlling for age and sex (*p* = 0.013, 12 edges, 13 nodes, Fig. 5b). Supplementary Table 1 includes a list of all connections identified in the network. Figure 5c shows the average Pearson Correlation Coefficients identified in each significant connection within each group.

Post-hoc analysis identified a group by sex interaction (*p* < 0.001, 5 edges, 6 nodes). Supplementary Table 2 contains a list of all connections identified in this network.

**Fig. 6:**
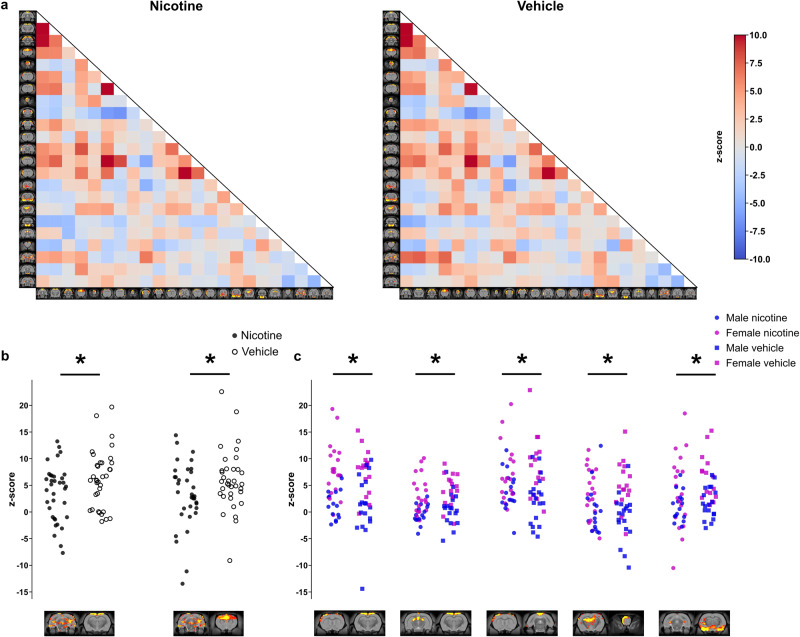
Nicotine-exposed animals showed decreased between component group connectivity. **a** Between-Resting state network average group connectivity. The values were calculated by averaging correlation coefficient within each group between the resting state networks resulting from the ICA. The results are shown as z-scores. **b** Significant differences in correlation strength were observed between nicotine and vehicle-exposed rats. Non-parametric permutation test was used (10,000 permutations). comparisons are adjusted for sex. A decrease in functional connectivity in animals exposed to nicotine vapour was seen between the hippocampus and the somatosensory cortex (left) components, and between the hippocampus and cingulate cortex (right) components. **c** Effects of sex on resting state network connectivity strength. Nodes showing significant interaction between sex and connectivity strength (ICA component pair-wise comparisons from left to right): Somatosensory and motor cortex, Hippocampus and somatomotor cortex, Somatosensory and default mode network, Hippocampus and olfactory bulb, Hippocampus and amygdala. FWER-corrected comparisons are presented. *p < 0.05.

Like the NBS analysis, between-component group analysis confirmed an overall reduction in connectivity due to nicotine vapour exposure, with reductions in connectivity between the hippocampus and somatosensory cortex (*p* = 0.02) and hippocampus and cingulate cortex (*p* = 0.041), as presented in Fig. 6b. Significant interactions between sex and functional connectivity were also seen between somatosensory and motor cortex (*p* = 0.0001), Hippocampus and somatomotor cortex (*p* = 0.0001), somatosensory and default mode network (*p* = 0.0016), Hippocampus and olfactory bulb (*p* = 0.0056), and Hippocampus and amygdala (*p* = 0.012), as shown in Fig. 6c.

No statistically significant changes were seen in structural connectivity through diffusion network-based analysis (*p* = 0.19). Supplementary Fig. 3 shows a representative fibre orientation distribution.

A summary of all behavioural, pharmacokinetic, and network connectivity is provided in Table 2.

**Table 2:** Summary of nicotine vapour effects on behaviour, pharmacokinetics, and network connectivity. Data expressed as mean ± SEM.

Measure	Age	Sex	Finding		
Place Conditioning	Adult	Male	No CPP at 600 s exposure		
		Female	CPP at 480 and 600 s exposures		
	Adolescent	Male	No CPP at 600 s exposure		
		Female	No CPP		
Withdrawal symptoms	Adult	Male	Withdrawal		
		Female	No withdrawal		
	Adolescent	Male	Withdrawal		
		Female	No withdrawal		
Locomotion	Adult	Male	Increased locomotion		
		Female	No effect on locomotion		
	Adolescent	Male	Increased locomotion		
		Female	No effect on locomotion		
Weight	Adult	Male	No effect on weight gain		
		Female	Decreased weight gain		
	Adolescent	Male	Decreased weight gain		
		Female	No effect on weight gain		
Nicotine Pharmaco-kinetics (Plasma; ng/mL)	**Time point**		**10 min (venous)**	**30 min (arterial)**	**60 min (arterial)**
	Adult	Male	152.0 ± 30.1	106.0 ± 18.2	96.5 ± 11.1
		Female	287.7 ± 29.5	195.7 ± 26.1	178.5 ± 16.7
	Adolescent	Male	175 ± 38.1	126.9 ± 16.9	82.2 ± 15.5
		Female	145 ± 36.8	177.8 ± 26.1	127.0 ± 30.1
Nicotine Pharmaco-kinetics (Brain; ng/g)	**Time point**		**10 min**	**30 min**	**60 min**
	Adult	Male	-	193.6 ± 30.6	93.6 ± 12.5
		Female	-	514.0 ± 77.5	333.5 ± 26.5
	Adolescent	Male	-	259.3 ± 31.2	192.0 ± 33.5
		Female	-	561.1 ± 62.6	397.7 ± 75.0
Functional Connectivity	Decreased NBS and between component group connectivity in nicotine vapour exposed animals; significant interaction with sex, with females showing further reductions in connectivity (NBS)				
Structural Connectivity	No effect on diffusion (NBS)				

## Discussion

In these experiments we investigated the differential sex- and age-dependent effects of JUUL e-cigarette nicotine vapour exposure on nicotine reward, withdrawal, pharmacokinetics, and brain connectivity. Our findings (summarised in Table 2) support the notion that nicotine’s actions vary as a function of age and sex.

### Place conditioning

Our present study found female adolescents to be less vulnerable to nicotine vapour’s reward-like properties than female adults which contrast our previous findings in males [28]. We also found that at the highest nicotine dose, vapour was no longer rewarding for any group except female adults. This is consistent with previous studies showing a right-shifted dose-response curve in female rodents [36, 39]. Female adolescents did not acquire CPP at any dose tested. Adolescent female mice have been found to have narrower dose ranges for CPP compared to adult females [61]; as such, the dose for CPP in female adolescents may be intermediate to those in the present experiment. Furthermore, our two investigations differed in JUUL flavour (mango vs. mint). Interestingly, the high concentrations of plasma and brain nicotine achieved via the use of vapourized nicotine salts in the present study, similar to those seen in a previous vapour self-administration study [43], if achieved via subcutaneous or intraperitoneal injections, would have produced aversive responses [62], suggesting that route of administration impacts the rewarding or aversive profiles of nicotine, as has also previously been suggested with THC [63]. A similar mismatch in dose and behaviour between routes of administration has also been seen with methamphetamine; for locomotor effects to be the same between exposure routes, rats needed ~10-fold higher plasma methamphetamine levels via vapour exposure compared to intraperitoneal injection [64], again highlighting the importance of considering routes of administration in the design of behavioural pharmacology studies.

### Withdrawal

Adult and adolescent males displayed similar levels of withdrawal. These findings contrast with previous osmotic minipump studies that suggested adolescent rats experience decreased withdrawal [30, 31]. Growing evidence suggests that route and timing of administration has a significant effect on the age-dependent differences in nicotine withdrawal, with inhalation resulting in anxiety-like behaviour and spontaneous withdrawal following nicotine cessation in adolescent rats [65, 66]. These new findings are more consistent with clinical findings that suggest adolescents may experience similar or greater withdrawal than adults [67]. Female rats did not show precipitated nicotine withdrawal. This is consistent with a study finding female rats to show no somatic signs of precipitated withdrawal [40], however, studies have found robust spontaneous withdrawal in females [40, 68]. The higher nicotine levels in females may have also increased nicotine availability during withdrawal assessment, thereby masking the precipitation of withdrawal by mecamylamine and delaying the onset of spontaneous withdrawal. It is additionally likely that nicotine withdrawal in females is under-estimated via currently existing methods, prompting the need for alternative methods of assessment (e.g., a global scoring system) [69]. While somatic withdrawal did not reach significance in adolescent nicotine females compared to vehicle, they did trend toward significance (t(51) = 1.989, *P* = 0.0521), supporting previous work in mice that found increased withdrawal in adolescent female mice compared to adults [61]. Further discussion on locomotion, weight, and the potential influence of oestrous and gonadal hormones on our behavioural findings are included in the Supplementary discussion.

### Pharmacokinetics

Nicotine vapour exposures resulted in the plasma nicotine range of 100–300 ng/ml. We found higher levels of nicotine and cotinine levels in adult females compared to adolescent females, but no age difference in male plasma or the brain supernatant of males or females contrasting previous studies showing lower brain and plasma nicotine and cotinine and higher nornicotine in adolescents compared to adults following subcutaneous or intravenous injection [20, 70]. This further supports a route-dependent differential nicotine distribution.

Adult females had significantly greater nicotine and cotinine plasma levels at the 10-min timepoint compared to adolescent females. Given our findings that females only find nicotine rewarding at higher doses, and previous clinical findings showing that women use higher nicotine concentrations when vaping [10], the lower plasma levels in adolescent females may be responsible for their lack of CPP. Importantly, the plasma levels achieved in our study are consistent with plasma levels previously shown in rats self-administering nicotine vapour [43]. Additional considerations effecting pharmacokinetics, such as hormonal influence and device and vehicle characteristics are discussed in the Supplementary discussion.

### Imaging

Functional MRI network analysis with NBS confirmed a single network that had significantly reduced connectivity in nicotine-treated rats, consistent with regions previously shown to have reduced connectivity in smokers [71–74]. Four of the 12 edges identified with NBS were connected to the hypothalamus (parietal cortex, primary and secondary visual cortex, retrosplenial cortex, and cornu ammonis 1). Orexin neurons in the hypothalamus are activated by contextual stimuli associated with multiple drugs of abuse [75], and blocking the orexin 1 receptor with the antagonist SB-334867 reduce nicotine self-administration [76]. This is particularly interesting, as three of the four altered connections with the hypothalamus were significantly more dysfunctional in females. This could explain why women and female rodents may be more sensitive to non-pharmacological nicotine cues, especially given the sensory nature of the connections [5]. Sensory structures have previously been found to be activated by nicotine exposure when measured by c-Fos expression in rats but have never been compared between sexes [77, 78]. The further decreased functional connectivity seen in females in the present study is consistent with the higher nicotine levels in our study. The potential disconnect between the behavioural and neural findings may relate to the acute effects of nicotine and potential greater receptor desensitisation in females due to the higher nicotine levels during behavioural assessments, which were no longer present at the time of the scans [79]. Additional discussion of reduced hypothalamic connectivity on our behavioural findings is included in the supplemental discussion.

Another edge of interest is the reduced functional connection between the insula and the cingulate as decreased resting state functional connectivity between these regions in humans has been associated with more severe nicotine dependence [80]. The same study found that a nicotine challenge did not significantly alter the circuit dysfunction, suggesting the dysfunction to reflect an addiction trait rather than pharmacological state. Together with our findings that this dysconnectivity can be acquired following chronic nicotine vapour exposure, this suggests the acquisition of the addictive trait could be the result of prolonged nicotine exposure rather than innate. This is consistent with a previous study investigating the causal directionality of cingulate-striatal dysfunction and its trans-species relationship with nicotine dependence severity; rats showed dysregulation of the circuit following 14 days of nicotine exposure via osmotic minipump which moderated intensity of precipitated withdrawal [81]. Together, these findings point to multiple nicotine-induced circuit dysfunctions centred on the insula that are associated with increased nicotine dependence, highlighting the insula as a core component in nicotine addiction neurophysiology and a primary target for treatment.

Additional between-component group analysis confirmed significant reductions in connectivity between the hippocampus and the somatosensory cortex, and between the hippocampus and cingulate cortex in the nicotine-treated group compared to vehicle. This finding could be a result of the neurotoxic effects of nicotine in the hippocampus. Previous results in mice have associated chronic nicotine exposure with reduced dendritic length in the basal tree of hippocampal pyramidal cells [82]. Similarly, clinical studies have shown smokers to have reduced hippocampal volume with MRI [83], and reduced hippocampal neuronal integrity with proton magnetic resonance spectroscopy [84]. Similar to NBS findings, an effect of age was not seen with the between-component group analysis. A large sex interaction was also observed as shown in Fig. 6c, however, the overall effect of nicotine was less clear than with the NBS analysis as these differences appeared to be largely driven by innate sex differences in connectivity.

### Limitations

There are several limitations to this study that should be considered when interpreting the findings. Due to JUULs proprietary nature, it was not possible to create a flavourless JUUL pod to fully dissociate the effects of flavour and nicotine on behaviour and thus eliminate flavour as a confound in this study. Despite our inclusion of mint flavouring in the vehicle pod, it is possible that the interaction between nicotine and flavourant could have contributed to our findings; this becomes especially relevant in the context of sex differences with a recent study showing that menthol enhanced self-administration of high-dose nicotine (60 mg/ml) vapour only in female, but not male, mice [85]. Furthermore, in the locomotor experiment, females had higher baseline activity, thus the lack of locomotor effect seen in the females could be the result of a ceiling effect. Moreover, as the behavioural, pharmacokinetic, and neuroimaging studies were not run in parallel, there is the potential for a cohort effect, which could, theoretically account for some of the differences seen.

The overall nicotine levels in this study were very high compared to those seen in IVSA and human smokers. It is thus surprising that adult female rats acquired CPP under these conditions. This mismatch in dose and behaviour between exposure routes is interesting and has been seen in other studies [43, 63], thus it is a result that should be investigated further. Additionally, a previous study comparing passive exposure to JUUL vapour (nicotine salt e-liquid) compared to Marlboro Red combustible cigarette or a tank-based device (freebase nicotine e-liquid) resulted in 6–8 times the serum nicotine levels 20 min after the exposure [16], thus salt-based devices may be resulting in much higher serum nicotine levels than those seen in smokers. Further studies should delineate the unique characteristics of nicotine salt and nicotine freebase to understand how these different formulations may be affecting behaviour and pharmacokinetics. Lastly, because our pharmacokinetics and MRI studies were run in parallel, the pharmacokinetic findings were not able to inform our neuroimaging study. Thus, we could not match nicotine levels between the sexes to effectively delineate effects of matching exposures at this time; however, these results will inform our future investigations.

## Conclusion

Overall, our findings suggest that the effects of nicotine vapour are differentially affected by both sex and age. While our previous findings found male adolescents to be more sensitive to nicotine vapour’s rewarding effects compared to adults, our current results show the opposite in females. Interestingly, despite higher plasma and brain nicotine concentrations, we also see no effects of nicotine on locomotion or withdrawal in females. Our fMRI findings show significant overlap with previous clinical and preclinical studies, especially in the insular and cingulate cortices with a greater impact in female rats exposed to nicotine vapour, particularly in hypothalamic connectivity. These findings taken together show that like humans, JUUL vapour can produce reward and dependence in a sex- and dose-dependent manner, as well as sex-dependent long-lasting changes in brain circuitry relevant to addiction.

### Supplementary information

Supplementary methods, data and discussion
