# Supplementary material for: Factors influencing JUUL e-cigarette nicotine vapour-induced reward, withdrawal, pharmacokinetics and brain connectivity in rats: sex matters
Source: Neuropsychopharmacology. 2023 Dec 7;49(5):782–95. doi: 10.1038/s41386-023-01773-3 (PMC10948865; doi:10.1038/s41386-023-01773-3)
Supplement: Supplementary file 1 — Supplementary methods, data and discussion [file 41386_2023_1773_MOESM1_ESM.pdf]

Supplementary Materials

FACTORS INFLUENCING JUUL NICOTINE VAPOUR REWARD, WITHDRAWAL, PHARMACOKINETICS AND BRAIN CONNECTIVITY IN RATS: SEX MATTERS

Jude A. Frie, BScH,[1] Patrick McCunn, PhD,[2] Amr Eed, PhD,[3] Ahmad Hassan,[1] Karling R. Luciani, BScH,[4] Chuyun Chen, MSc[1] Rachel F. Tyndale, PhD,[5] Jibran Y. Khokhar, PhD[1,2]

[1] Department of Biomedical Sciences, Ontario Veterinary College, University of Guelph, Guelph, Ontario, Canada.

[2] Department of Anatomy and Cell Biology, Schulich School of Medicine and Dentistry, Western University, London, Ontario, Canada.

[3] Department of Medical Biophysics and Robarts Research Institute, Schulich School of Medicine and Dentistry, Western University, London, Ontario, Canada.

[4] Department of Psychiatry, Faculty of Medicine, University of British Columbia, Vancouver, British Columbia, Canada.

[5] Departments of Psychiatry, and Pharmacology & Toxicology, University of Toronto, Campbell Family Mental Health Research Institute, Centre for Addiction and Mental Health, Toronto, Canada.

Corresponding Author: Jibran Khokhar
Telephone: 519.661.2111 Ext. 81524
Email: jkhokha@uwo.ca
Address: Room 472, Medical Sciences Building, 1151 Richmond St, London, ON, N6A 3K7

SUPPLEMENTAL METHODS

Functional

The processing of fMRI images was conducted using the open-source RABIES software [1]. For both the anatomical and functional images, extra-space around the brain was automatically cropped and temporal spikes were corrected for at each voxel [2]. Dummy scans were automatically detected and removed from each EPI. If dummy scans are detected, the median of these volumes provides a volumetric EPI image as reference, given their higher anatomical contrast. Otherwise, a volumetric EPI image was derived using a trimmed mean across the EPI frames, after an initial motion realignment step. Using this volumetric EPI as a target, the head motion parameters were estimated by realigning each EPI frame to the target using a rigid registration. To conduct common space alignment, structural images, were corrected for inhomogeneities, and then registered together to allow the alignment of different MRI acquisitions. This registration was conducted by generating an unbiased data-driven template through the iterative nonlinear registration of each image to the dataset consensus average, where the average gets updated at each iteration to provide an increasingly representative dataset template [3]. The finalized template after the last iteration provides a representative alignment of each MRI session to a template that shares the acquisition properties of the dataset, which makes it a stable registration target for cross-subject alignment. After aligning the MRI sessions, this newly generated unbiased template was then itself registered, using a nonlinear registration, to the SIGMA rat brain template [4]. To correct for EPI susceptibility distortions, the volumetric EPI was also subjected to inhomogeneity correction, and then registered using a nonlinear registration to the anatomical scan from the same MRI session [5].

Finally, after calculating the transformations required to correct for head motion and susceptibility distortions, transforms were concatenated into a single resampling operation (avoiding multiple resampling) which is applied at each EPI frame, generating the preprocessed EPI timeseries in native space [6]. Preprocessed timeseries in common space were also generated by further concatenating the transforms allowing resampling to the reference atlas, at a voxel resolution of 0.3x0.3x0.3mm.

Confound correction was executed on the EPI timeseries resampled to commonspace. Voxelwise linear detrending was first applied to remove first-order drifts and the average image. Motion sources were then automatically removed using a modified version of the ICA-AROMA classifier where classifier parameters and anatomical masks are instead adapted for rodent images [7]. The original ICA-AROMA algorithm, which was designed for human data, was adapted to work with rodent data by modifying the hard-coded human priors for anatomical masking and parameter thresholds for component classification. After an initial independent component analysis (ICA) decomposition of the data, four features are extracted from each ICA component spatial map for classification. The component is classified as motion if the CSF or high frequency content fractions are above a given threshold, or if classified by a pre-trained linear classifier combining the brain edge fraction and motion correlation. The CSF mask is inherited from the rodent reference atlas and the edge mask is automatically generated from the brain mask. The threshold for high frequency content was increased as rodents can express higher BOLD frequencies, particularly under medetomidine. The linear classifier was retrained to select new parameters. The automatic process was evaluated by manually classifying motion and network components, derived from a set of scans from the REST-AWK group anesthetized under a medetomidine-isoflurane mixture, and selected parameters to successfully classify clear motion components while avoiding false classification of brain networks. Next, low pass filtering (0.1Hz) and high pass filtering (0.01Hz) was applied [8]. Estimated nuisance time-courses during preprocessing were then used for

confound regression. More specifically, using ordinary least square regression, the 6 rigid motion parameters, the mean signal from the WM and CSF masks and the global signal were modelled at each voxel and regressed from the data. Before analysis, a spatial Gaussian smoothing filter was applied at 0.3mm full-width at half maximum (FWHM) [8].

Whole-brain connectivity matrices were generated in commonspace for each subject individually using the SIGMA functional template (59 Regions of Interest) by extracting the seed time-course for every parcel and then measuring the cross-correlation (Pearson's r) between every region pair. The correlation values were then re-organized into a whole-brain matrix representing the 'connectivity strength' between every corresponding region pair.

Diffusion

Images were preprocessed using the fMRI Software Library (FSL, v. 6.0.4) and MRtrix (v. 3.0.2) [9]. Gibbs Ringing Removal [10], followed by PCA denoising [11], was performed first in MRtrix. TOPUP [12,13], followed by EDDY [14], was used to correct for eddy current induced distortions as well as susceptibility-induced distortions. Tractography was then performed using the MRtrix software package. Response functions for single-fibre WM as well as GM and CSF were estimated from the data themselves using an unsupervised method [15]. Fibre orientation distribution images were calculated using multi-tissue spherical deconvolution (msmt_csd) followed by images undergoing multi-tissue informed log-domain intensity normalization [16,17]. Whole brain tractograms were generated using second-order Integration over Fiber Orientation Distributions (iFOD2) with 10 million streamlines [18], followed by filtering of tractograms [19].

Diffusion data was registered in the same way as the functional data above with one difference: inverse transformation matrices were used to bring the SIGMA atlas regions-of-interest into diffusion space for final analysis. This was done due to the unique spatial nature of diffusion imaging, and the potential for confounding effects due to resampling and registration of the diffusion data to commonspace. Finally, using the SIGMA ROIs in diffusion space, single subject connectomes weighted by Streamline Count (SC) were produced.

Statistical Analysis

Network-based statistic (NBS) was used to identify significantly different subnetworks (clusters of nodes and edges) between groups [20]. Briefly, NBS first identifies edges that surpass a given threshold (suprathreshold links), followed by identification of connected nodes within this subnetwork and finally permutation testing to assign a p-value (controlled for the FWE) to each subnetwork based on its size. Specifically, a test statistic and corresponding p-value is independently computed for each link based on the strength of the pairwise association the link represents. Two tailed t-tests were run to test for main effects of group ($p = .05$, t-threshold = 3.1) with age and sex introduced to the GLM as a covariate. Statistically significant networks ($p < .05$) were extracted for analysis of interaction effects. Within these statistically significant networks, the group by age interaction and the group by sex interaction were tested using an f-test ($p=.025$, f-threshold = 5).

SUPPLEMENTAL DISCUSSION

Locomotion

We found that repeated passive nicotine vapour exposure resulted in hyperlocomotion in both adult and adolescent males, but not females. These results match a recent study that compared intravenous nicotine to nicotine vapour; in contrast to i.v. nicotine that produced hyperlocomotion in both male and female rats, passive nicotine vapour only produced hyperlocomotion in males [21]. Interestingly, these findings counter several studies that find females are more sensitive to nicotine hyperlocomotion following chronic exposure via other

routes, suggesting a unique behavioural result associated with nicotine vapour inhalation [22–24]. Females did, however, have higher locomotion at baseline; therefore, the lack of hyperlocomotion seen could be a result of a potential ceiling effect. It is likely that the difference in locomotion is a result of differing nicotine levels in the blood as shown by our pharmacokinetic results. Given locomotion's inverted U-shaped curve in response to nicotine [25], the females may be in the latter half of the curve where nicotine is beginning to have a suppressing effect on locomotion.

Weight

Adult females were more sensitive to nicotine's suppression of weight gain compared to adult males as seen previously [26]. Also consistent with previous findings, adolescent males were more sensitive to nicotine's suppression of weight gain compared to adolescent females [27]. Thus, the effects of nicotine on weight appear to be reliable across exposure routes.

Influence of Estrous and Gonadal Hormones on Behavioural Findings

It is unlikely that the sex differences in nicotine reward, withdrawal, or locomotion observed in the present findings are the result of estrous, as previous studies have found no effect of estrous phase on these behaviours [28–32]. Additionally, the low variability in plasma and brain levels seen within females in our pharmacokinetics results suggest that hormonal differences did not lead to large changes in plasma nicotine or metabolite levels. One study did find that tamoxifen co-administration facilitated CPP in female rats [33], and another found that ovariectomy eliminated nicotine induced CPP [34]; gonadal hormones are thus likely important factors in nicotine reward but the fluctuations during estrous are not enough to cause significant variations in nicotine reward at the sample sizes used in these experiments. It is thus more probable that sex dependent behavioural differences are the result of organizational hormonal effects, as well as overall differences in average gonadal hormone levels. Differences could also result from altered nicotinic acetylcholine receptor (nAChR) response and expression. There are known sex differences in nAChR upregulation in response to chronic nicotine; males show a greater nAChR upregulation which has been seen in mice [35], rats [36], and humans [37].

Additional Pharmacokinetic Considerations

A previous study found that adult female rats given repeated i.v. nicotine resulted in a >10-fold nicotine plasma concentration compared to males; this difference was attenuated when males were castrated and females were gonadectomized, indicating that gonadal hormones influence nicotine pharmacokinetics [38]. In line with this finding, our study found adult females had greater nicotine and nicotine metabolite concentrations than adult males at every time point in both blood plasma and brain supernatant. This was similar in adolescent female brain supernatant but not plasma. Previous studies on vapour pharmacokinetics have been inconsistent; one study found reduced cotinine plasma in females following passive nicotine vapour exposure compared to males [21], while another found increased cotinine in only female adolescents [39]. These differences may be a result of the inconsistent power settings used throughout the literature; lower wattage during vapourization results in smaller particle size, leading to significantly increased respirable fraction of aerosol [40]. Because larger particles have a lower probability of entering the lungs, it is likely that the size difference in the oropharyngeal cavity between male and female rats results in decreased deposition of large nicotine vapour particles in female or adolescent rats' lungs compared to those of males or adults respectively. It therefore may be important to use low power devices in future experiments when comparing animals that differ in size. Additional variables in the literature such as PG:VG ratio, vapourizer temperature, ohmage, exposure duration, nicotine type (salt

or base), and rat strain also likely contribute to varied findings. There were no sex differences in adolescent nicotine plasma levels as has been observed following tobacco smoke exposure or s.c. nicotine injection previously [41].

Hypothalamic Influence on Behaviour

Dysfunction in hypothalamic connectivity could also explain the lack of withdrawal observed in the present study as well as sex differences in the long-term effects of adolescent JUUL vapour exposure on sign-tracking in adulthood seen previously in our lab [42]. Orexin signaling in the hypothalamic paraventricular nucleus has been shown to be important for the expression of nicotine withdrawal [43]. The decreased hypothalamic connectivity seen in females could therefore be inhibiting their ability to show withdrawal-like symptoms. We have also previously found that males but not females demonstrated enhanced levels of sign-tracking behaviour when previously exposed to chronic nicotine vapour in adolescence [42]. Sign- trackers show increased cue-induced activity in the hypothalamus [44]; thus, if this region shows dysconnectivity following chronic nicotine exposure, females could be resilient to adolescent nicotine-induced sign-tracking as a result.

SUPPLEMENTARY TABLES AND FIGURES

Supplementary Table 1: All connections identified in the Nicotine group, to have statistically significantly reduced functional connectivity through NBS statistics (p=0.013, 12 edges, 13 nodes).

Edge	Node	Name	Hemi-sphere	Territory	Node	Name	Hemisphere	Territory	Test Stat
**1**	12	Primary Somatosensory Cortex	Left	Cortex	16	Endo/Piriform Cortex	Left	Cortex	3.74
**2**	11	Primary Somatosensory Cortex	Right	Cortex	17	Insular Cortex 2	Right	Cortex	3.31
**3**	17	Insular Cortex 2	Right	Cortex	18	Cingulate Cortex 3	Interhemi-spheric	Cortex	3.72
**4**	21	Hypothalamus 1	Interhemispheric	Dience-phalon	29	Parietal Cortex (Auditory)	Left	Cortex	3.44
**5**	16	Endo/Piriform Cortex	Left	Cortex	39	Primary and Secondary Visual Cortex	Left	Cortex	3.63
**6**	17	Insular Cortex 2	Right	Cortex	39	Primary and Secondary Visual Cortex	Left	Cortex	3.73
**7**	21	Hypothalamus 1	Interhemispheric	Dience-phalon	39	Primary and Secondary Visual Cortex	Left	Cortex	3.27
**8**	16	Endo/Piriform Cortex	Left	Cortex	40	Dorsal Dentate Gyrus	Left	Cortex	3.25
**9**	21	Hypothalamus 1	Interhemispheric	Dience-phalon	41	Retrosplenial Cortex 3/Superior Gray	Interhemi-spheric	Cortex	3.25
**10**	21	Hypothalamus 1	Interhemispheric	Dience-phalon	48	Cornu Ammonis 1 (Ventral)	Left	Cortex	3.70
**11**	22	Piriform Cortex	Right	Cortex	48	Cornu Ammonis 1 (Ventral)	Left	Cortex	3.15
**12**	16	Endo/Piriform Cortex	Left	Cortex	57	Colliculus	Right	Mesence-phalon	3.28

Supplementary Table 2: All connections identified by NBS statistics to have statistically significant interaction Group by Sex effect ($p < 0.001$, 5 edges, 6 nodes).

**Edge**	**Node**	**Name**	**Hemi-sphere**	**Territory**	**Node**	**Name**	**Hemisphere**	**Territory**	**Test Stat**
**4**	21	Hypothalamus 1	Interhemispheric	Diencephalon	29	Parietal Cortex (Auditory)	Left	Cortex	8.16
**5**	16	Endo/Piriform Cortex	Left	Cortex	39	Primary and Secondary Visual Cortex	Left	Cortex	9.66
**7**	21	Hypothalamus 1	Interhemispheric	Diencephalon	39	Primary and Secondary Visual Cortex	Left	Cortex	9.57
**10**	21	Hypothalamus 1	Interhemispheric	Diencephalon	48	Cornu Ammonis 1 (Ventral)	Left	Cortex	13.48
**11**	22	Piriform Cortex	Right	Cortex	48	Cornu Ammonis 1 (Ventral)	Left	Cortex	12.28

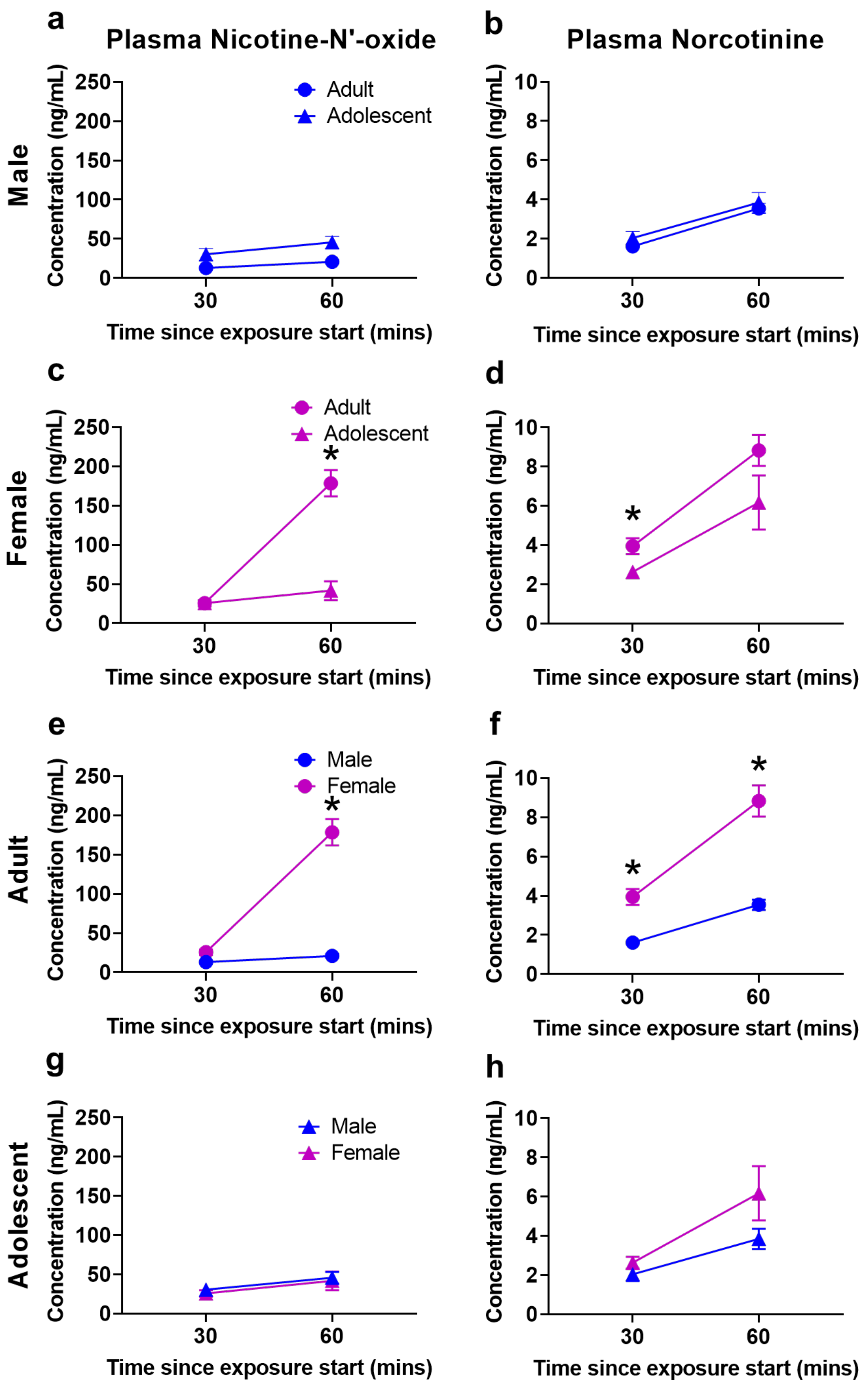

Supplemental Figure 1: Arterial plasma nicotine-N'-oxide and norcotinine concentrations 30, and 60 minutes after 10 minutes of JUUL vapour exposure. **a) & b)** Comparison of adult males to adolescent males. **c) & d)** Comparison of adult females to adolescent females. Female adults have higher plasma nicotine-N'-oxide at 60 minutes and greater norcotinine at 30 minutes compared to adolescents. **e) & f)** Comparison of adult male to adult females. Adult females have higher nicotine-N'-oxide at 60 minutes and greater norcotinine at 30 and 60 minutes compared to adult males. **g) & h)** Comparison of adolescent male to adolescent female plasma concentrations. ***p<0.05** adult versus adolescent or male versus female. N=7 per group and timepoint. Data presented as **mean ± SEM**.

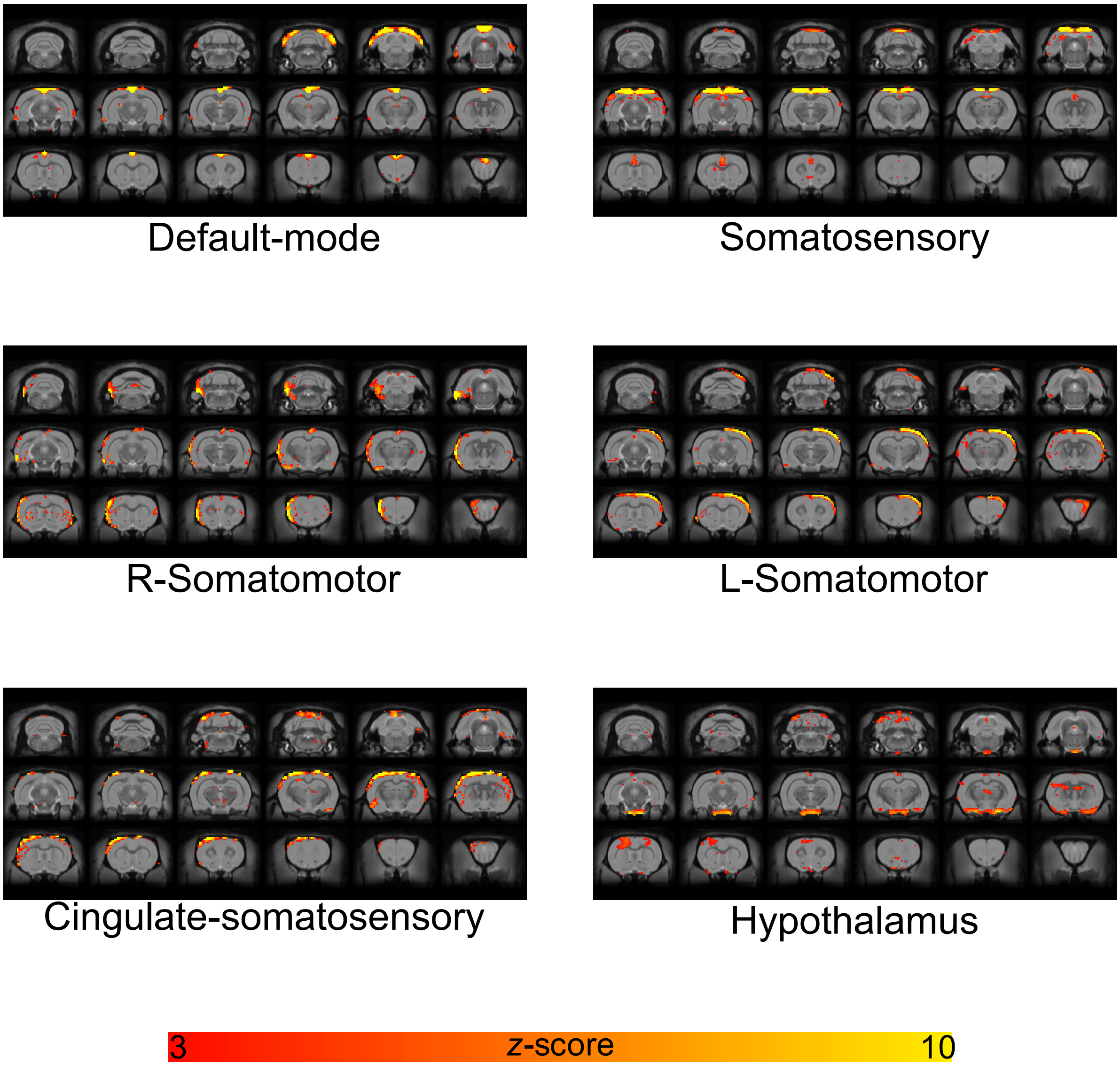

Supplementary Figure 2: Common resting-state networks identified using spatial group-level ICA. The images are shown in an anatomical view overlaid on a commonspace anatomical template, resampled to the EPI's dimensions and the networks are scaled in pseudo-z-scores, i.e., how many standard deviations away from the background noise.

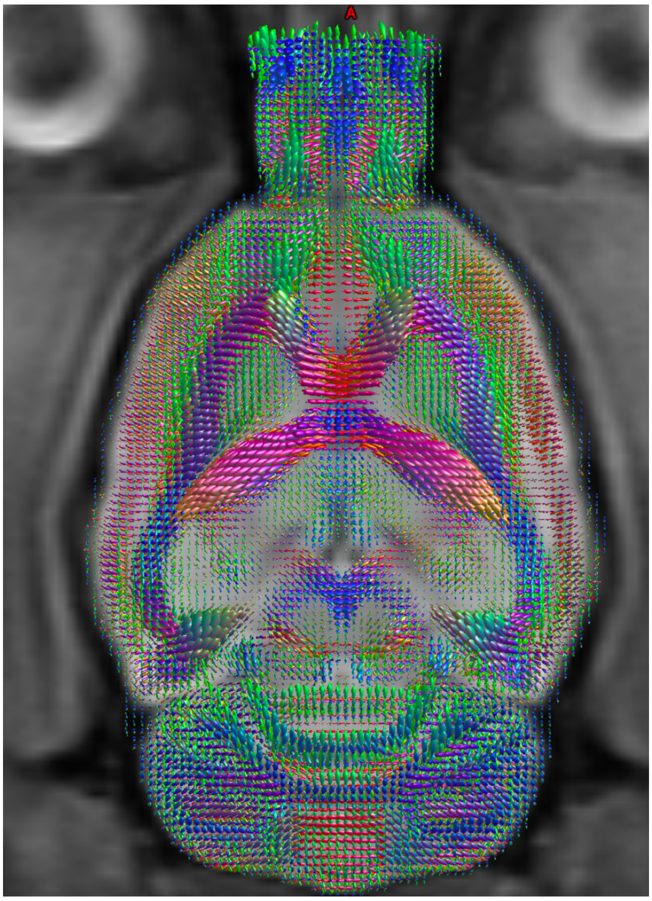

Supplementary Figure 3: Representative Fibre Orientation Distributions (FODs) calculated using multi-tissue spherical deconvolution (msmt_csd) which were used to generate whole brain tractograms second-order Integration over Fiber Orientation Distributions.

References

1. Desrosiers-Gregoire G, Devenyi GA, Grandjean J, Chakravarty MM. Rodent Automated Bold Improvement of EPI Sequences (RABIES): A standardized image processing and data quality platform for rodent fMRI. Biorxiv. 2022:2022.08.20.504597.

2. Cox RW. AFNI: Software for Analysis and Visualization of Functional Magnetic Resonance Neuroimages. Comput Biomed Res. 1996;29:162–173.

3. Avants BB, Tustison NJ, Song G, Cook PA, Klein A, Gee JC. A reproducible evaluation of ANTs similarity metric performance in brain image registration. Neuroimage. 2011;54:2033–2044.

4. Barrière DA, Magalhães R, Novais A, Marques P, Selingue E, Geffroy F, et al. The SIGMA rat brain templates and atlases for multimodal MRI data analysis and visualization. Nat Commun. 2019;10:5699.

5. Wang S, Peterson DJ, Gatenby JC, Li W, Grabowski TJ, Madhyastha TM. Evaluation of Field Map and Nonlinear Registration Methods for Correction of Susceptibility Artifacts in Diffusion MRI. Front Neuroinform. 2017;11:17.

6. Esteban O, Markiewicz CJ, Blair RW, Moodie CA, Isik AI, Erramuzpe A, et al. fMRIPrep: a robust preprocessing pipeline for functional MRI. Nat Methods. 2019;16:111–116.

7. Pruim RHR, Mennes M, Rooij D van, Llera A, Buitelaar JK, Beckmann CF. ICA-AROMA: A robust ICA-based strategy for removing motion artifacts from fMRI data. Neuroimage. 2015;112:267–277.

8. Abraham A, Pedregosa F, Eickenberg M, Gervais P, Mueller A, Kossaifi J, et al. Machine learning for neuroimaging with scikit-learn. Front Neuroinform. 2014;8:14.

9. Tournier J-D, Smith R, Raffelt D, Tabbara R, Dhollander T, Pietsch M, et al. MRtrix3: A fast, flexible and open software framework for medical image processing and visualisation. NeuroImage. 2019;202:116137.

10. Kellner E, Dhital B, Kiselev VG, Reisert M. Gibbs-ringing artifact removal based on local subvoxel-shifts. Magn Reson Med. 2016;76:1574–1581.

11. Veraart J, Novikov DS, Christiaens D, Ades-aron B, Sijbers J, Fieremans E. Denoising of diffusion MRI using random matrix theory. NeuroImage. 2016;142:394–406.

12. Smith SM, Jenkinson M, Woolrich MW, Beckmann CF, Behrens TEJ, Johansen-Berg H, et al. Advances in functional and structural MR image analysis and implementation as FSL. NeuroImage. 2004;23:S208–S219.

13. Andersson JLR, Skare S, Ashburner J. How to correct susceptibility distortions in spin-echo echo-planar images: application to diffusion tensor imaging. NeuroImage. 2003;20:870–888.

14. Andersson JLR, Sotiropoulos SN. An integrated approach to correction for off-resonance effects and subject movement in diffusion MR imaging. NeuroImage. 2016;125:1063–1078.

15. Dhollander T, Raffelt D, Connelly A. Unsupervised 3-tissue response function estimation from single-shell or multi-shell diffusion MR data without a co-registered T1 image. ISMRM Workshop on Breaking the Barriers of Diffusion MRI, 2016. p. 6.

16. Tournier J-D, Calamante F, Gadian DG, Connelly A. Direct estimation of the fiber orientation density function from diffusion-weighted MRI data using spherical deconvolution. NeuroImage. 2004;23:1176–1185.

17. Jeurissen B, Tournier J-D, Dhollander T, Connelly A, Sijbers J. Multi-tissue constrained spherical deconvolution for improved analysis of multi-shell diffusion MRI data. NeuroImage. 2014;103:411–426.

18. Tournier J-D, Calamante F, Connelly A. Improved Probabilistic Streamlines Tractography by 2nd Order Integration Over Fibre Orientation Distributions. Proceedings of the International Society for Magnetic Resonance in Medicine, 2010. p. 1670.

19. Smith RE, Tournier J-D, Calamante F, Connelly A. SIFT2: Enabling dense quantitative assessment of brain white matter connectivity using streamlines tractography. NeuroImage. 2015;119:338–351.

20. Zalesky A, Fornito A, Bullmore ET. Network-based statistic: Identifying differences in brain networks. Neuroimage. 2010;53:1197–1207.

21. Lallai V, Chen Y, Roybal MM, Kotha ER, Fowler JP, Staben A, et al. Nicotine e-cigarette vapor inhalation and self-administration in a rodent model: Sex- and nicotine delivery-specific effects on metabolism and behavior. Addict Biol. 2021;26:e13024.

22. Kanýt L, Stolerman IP, Chandler CJ, Saigusa T, Pöğün Ş. Influence of Sex and Female Hormones on Nicotine-Induced Changes in Locomotor Activity in Rats. Pharmacol Biochem Be. 1999;62:179–187.

23. Harrod SB, Mactutus CF, Bennett K, Hasselrot U, Wu G, Welch M, et al. Sex differences and repeated intravenous nicotine: behavioral sensitization and dopamine receptors. Pharmacol Biochem Be. 2004;78:581–592.

24. Booze RM, Welch MA, Wood ML, Billings KA, Apple SR, Mactutus CF. Behavioral Sensitization Following Repeated Intravenous Nicotine Administration Gender Differences and Gonadal Hormones. Pharmacol Biochem Be. 1999;64:827–839.

25. Ksir C, Hakan RL, Kellar KJ. Chronic nicotine and locomotor activity: influences of exposure dose and test dose. Psychopharmacology. 1987;92:25–29.

26. Grunberg NE, Bowen DJ, Winders SE. Effects of nicotine on body weight and food consumption in female rats. Psychopharmacology. 1986;90:101–105.

27. Faraday MM, Elliott BM, Grunberg NE. Adult vs. adolescent rats differ in biobehavioral responses to chronic nicotine administration. Pharmacol Biochem Be. 2001;70:475–489.

28. Rao DN, Desiraju T. Comparative assessment of pedal pressing rates of self-stimulation of hypothalamus and midbrain with both square wave and sine wave stimulus parameters. Indian J Physiology Pharmacol. 1990;34:162–170.

29. Stratmann JA, Craft RM. Intracranial self-stimulation in female and male rats: no sex differences using a rate-independent procedure. Drug Alcohol Depen. 1997;46:31–40.

30. Hamilton KR, Berger SS, Perry ME, Grunberg NE. Behavioral effects of nicotine withdrawal in adult male and female rats. Pharmacol Biochem Be. 2009;92:51–59.

31. Tan S, Xue S, Behnood-Rod A, Chellian R, Wilson R, Knight P, et al. Sex differences in the reward deficit and somatic signs associated with precipitated nicotine withdrawal in rats. Neuropharmacology. 2019;160:107756.

32. Kuo DY, Lin TB, Huang CC, Duh SL, Liao JM, Cheng JT. Nicotine-induced hyperlocomotion is not modified by the estrous cycle, ovariectomy and estradiol replacement at physiological level. Chin J Physiology. 1999;42:83–88.

33. Yararbas G, Pogun S. Tamoxifen and mifepriston modulate nicotine induced conditioned place preference in female rats. Brain Res Bull. 2011;84:425–429.

34. Torres OV, Natividad LA, Tejeda HA, Weelden SAV, O'Dell LE. Female rats display dose-dependent differences to the rewarding and aversive effects of nicotine in an age-, hormone-, and sex-dependent manner. Psychopharmacology. 2009;206:303–312.

35. Mochizuki T, Villemagne VL, Scheffel U, Dannals RF, Finley P, Zhan Y, et al. Nicotine induced up-regulation of nicotinic receptors in CD-1 mice demonstrated with an in vivo radiotracer: Gender differences. Synapse. 1998;30:116–118.

36. Koylu E, Demirgören S, London ED, Pöğün S. Sex difference in up-regulation of nicotinic acetylcholine receptors in rat brain. Life Sci. 1997;61:PL185–PL190.

37. Cosgrove KP, Esterlis I, McKee SA, Bois F, Seibyl JP, Mazure CM, et al. Sex Differences in Availability of β2*-Nicotinic Acetylcholine Receptors in Recently Abstinent Tobacco Smokers. Arch Gen Psychiat. 2012;69:418–427.

38. Harrod SB, Booze RM, Mactutus CF. Sex differences in nicotine levels following repeated intravenous injection in rats are attenuated by gonadectomy. Pharmacol Biochem Be. 2007;86:32–36.

39. Espinoza VE, Giner P, Liano I, Mendez IA, O'Dell LE. Sex and age differences in approach behavior toward a port that delivers nicotine vapor. J Exp Anal Behav. 2022;117:532–542.

40. Floyd EL, Queimado L, Wang J, Regens JL, Johnson DL. Electronic cigarette power affects count concentration and particle size distribution of vaping aerosol. Plos One. 2018;13:e0210147.

41. Chellian R, Behnood-Rod A, Wilson R, Kamble SH, Sharma A, McCurdy CR, et al. Adolescent nicotine and tobacco smoke exposure enhances nicotine self-administration in female rats. Neuropharmacology. 2020;176:108243.

42. Ruffolo J, Frie JA, Thorpe HHA, Talhat MA, Khokhar JY. Alcohol and Vaporized Nicotine Co-exposure During Adolescence Contribute Differentially to Sex-Specific Behavioral Effects in Adulthood. Nicotine Tob Res. 2021;24:1177–1185.

43. Plaza-Zabala A, Flores Á, Maldonado R, Berrendero F. Hypocretin/Orexin Signaling in the Hypothalamic Paraventricular Nucleus is Essential for the Expression of Nicotine Withdrawal. Biol Psychiat. 2012;71:214–223.

44. Haight JL, Fuller ZL, Fraser KM, Flagel SB. A food-predictive cue attributed with incentive salience engages subcortical afferents and efferents of the paraventricular nucleus of the thalamus. Neuroscience. 2017;340:135–152.
